# Effects of Different Grazing Intensities on Soil C, N, and P in an Alpine Meadow on the Qinghai—Tibetan Plateau, China

**DOI:** 10.3390/ijerph15112584

**Published:** 2018-11-19

**Authors:** Gang Li, Zhi Zhang, Linlu Shi, Yan Zhou, Meng Yang, Jiaxi Cao, Shuhong Wu, Guangchun Lei

**Affiliations:** 1 School of Nature Conservation, Beijing Forestry University, Beijing 100083, China; ligang27@foxmail.com (G.L.); zhangzqh@126.com (Z.Z.); linlu.shi@foxmail.com (L.S.); zhouyan.eco@foxmail.com (Y.Z.); yangmeng@igsnrr.ac.cn (M.Y.); 2 Department of Landscape Architecture, School of Biology and Food Engineering, Fuyang Normal University, Fuyang 236037, China; 3 Fuzhou Planing Design & Research Institute, Urban Research Center, Fuzhou 350108, China; 4 Co-Innovation Center for Sustainable Forestry in Southern China, College of Biology and the Environment, Nanjing Forestry University, Nanjing 210037, China; 5 Key Laboratory of Ecosystem Network Observation and Modeling, Institute of Geographic Sciences and Natural Resources Research, Chinese Academy of Sciences, Beijing 100101, China; 6 School of Soil and Water Conservation, Beijing Forestry University, Beijing 100083, China; jason.apt@foxmail.com

**Keywords:** alpine meadow, grazing intensity, soil carbon, soil nitrogen, soil phosphorus

## Abstract

Inappropriate grazing management is one of the most common causes of grassland degradation, and thus, an assessment of soil properties under different grazing intensities is critical for understanding its effects on ecosystem nutrient cycling and for formulating appropriate management strategies. However, the responses of certain main elements, including soil carbon, nitrogen, and phosphorus, to grazing in alpine meadow ecosystems remain insufficiently clarified. Here, we measured carbon, nitrogen, and phosphorus contents in the topmost 30 cm of soil in an alpine meadow under three grazing intensities (light, moderate, and heavy) and found clear differences in soil physical and chemical properties among different grazing intensities and soil layers. As grazing intensity increased, soil water content, carbon and nitrogen contents and stocks, and carbon to phosphorus and nitrogen to phosphorus ratios decreased, whereas soil bulk density increased. However, soil phosphorus and carbon to nitrogen ratio remained stable. Our findings highlight the negative impacts of heavy grazing intensity, in terms of soil carbon and nitrogen loss and phosphorus mineralization. Moreover, we emphasize that further related studies are necessary to gain a more comprehensive understanding of the effects of grazing on grassland ecosystems, and thereby provide information for sustainable management practices and eco-compensation policies.

## 1. Introduction

Grazing in one of the dominant uses of grasslands, and via its effects on ecosystem nutrient cycling and services, grazing has the potential to render this type of ecosystem very fragile and sensitive to global climate change and anthropogenic interference [1,2,3,4], and also to promote the loss of stored soil elements [5,6,7]. One of the major issues relating to sustainability is degradation of the alpine grassland ecosystem caused by grazing. Numerous studies have examined the influence of grazing on the vegetation and soil properties of the alpine ecosystem [1,8,9] and, for example, have shown that grazing can enhance the diversity of vegetation communities and increase root to shoot ratios, but can also significantly decrease the amounts of carbon (C) stored in vegetation [10,11,12,13], above- and below-ground biomass [14,15], and soil C, nitrogen (N), and phosphorus (P) availability [16] and stocks [17]. Hence, overgrazing represents a significant anthropogenic pressure on pasture ecosystem stability and health. Vegetation and soil nutrient status (C, N, and P) can be the primary concern prompting an assessment of pasture degradation [18]. However, given the inconsistent results reported by different studies regarding the effects of grazing on certain major soil elements, the impact of grazing on soil properties could be far more complex than hitherto believed [13,19]. For example, some research has suggested that more intense grazing could be a better practical grazing strategy for sustainable grassland management [2,8], whereas other studies have shown that grazing results in a decrease in soil nutrient concentrations and biomass [1,5,20,21]. Consistently, it has been demonstrated that soil C, available N, and available P all increase following grazing exclusion [10,22], although there are other studies that have reported no significant change in soil nutrients between grazing exclusion and different grazing intensities [23,24]. These inconsistent results may be attributable to large differences in the duration and/or intensity of grazing, soil heterogeneity, variation in vegetational communities, and environmental conditions [3,25]. Hence, further studies are necessary to gain a more thorough understanding of how soils respond to grazing, which could yield important information for sustainable management practices and eco-compensation policies.

In addition to soil element concentrations, the stoichiometric variation of C, N, and P in soils and their biogeochemical cycling in terrestrial ecosystems have long been recognized as important factors with respect to ecological stability and nutrient limitation [26,27,28]. For example, high C:N ratios (>25 on a mass basis) indicate that organic matter accumulation is occurring faster than decomposition [29], whereas organic C to P ratios of <200, >300, and 200–300 can be indicative of the mineralization, immobilization, or stability of soil P, respectively [30,31]. The accumulation and stoichiometry of soil elements are controlled by the vegetation-soil-microbial nutrition cycle, which is influenced by both environmental conditions and management practices [31,32]. Compared with natural ecosystems, management practices such as grazing have a greater potential to perturb soil nutrient cycles [1,33]. Because the densities of livestock employed in grazing are typically greater than those of grazing animals under natural conditions, this tends to lead to grassland degradation, which in turn has certain repercussions with regards to soil properties [34]. Although previous studies have investigated the spatial distribution and ratios of C, N, and P in natural meadows on the Qinghai-Tibetan Plateau [35], little research has focused on the stoichiometric variation of C, N, and P in alpine meadows under different grazing intensities. Moreover, compared with the soil C:N ratio, the effects of grazing on stoichiometric variation in soil C:P and N:P ratios remain poorly understood [36,37].

In the Qinghai-Tibetan Plateau region of China, livestock grazing is the dominant form of land use in alpine grasslands, which has led to substantial changes in the character of the local alpine meadow ecosystem [3,38]. Considering the importance of the Qinghai-Tibetan Plateau as a global ecosystem, in terms of its large stocks of soil C, N, and P [39,40], it is imperative to gain a more complete understanding of the impact of grazing on the alpine meadows in this region. In particular, we need to determine the functional responses of vegetation and soil nutrients to grazing under different grazing management strategies. Hence, in the present study, we examined the responses of vegetation biomass and soil C, N, and P availability to different grazing intensities in the northeastern part of the Qinghai-Tibetan Plateau, with the specific aim of quantifying the impacts of three different grazing intensities on soil C, N, and P contents and stocks and their stoichiometric variation at 10-cm intervals in the topmost 30 cm of soil. Our guiding hypothesis in this study was that soil C, N, and P concentrations and stoichiometries would either increase or decrease in response to an increase in grazing intensity.

## 2. Materials and Methods

### 2.1. Study Area

**Figure 1:**
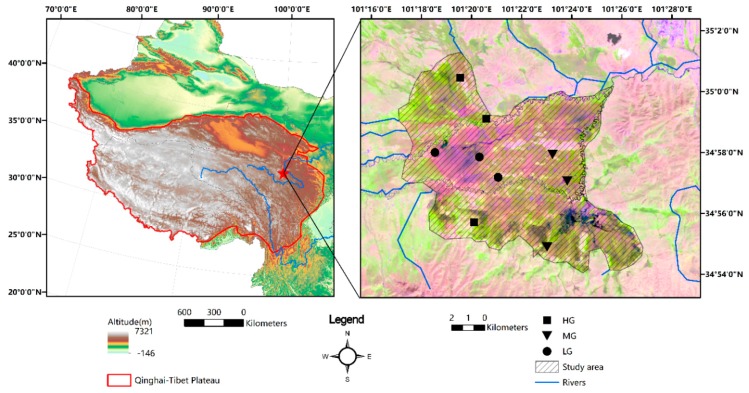
Location of the study area and sampling sites under three grazing intensities in the Qinghai-Tibetan Plateau. (LG, light grazing; MG, moderate grazing; HG, heavy grazing).

The present study was carried out in alpine meadow pasture in the northeastern region of the Qinghai-Tibetan Plateau (latitude 34°45′–35°32′, longitude 100°34′–102°08′; altitude 3700 m), located in Zeku County, Qinghai, China (Figure 1). The prevailing climate of the region is one of continental monsoon, with a mean annual temperature of −2.4 °C and mean annual precipitation of 437–511 mm. According to the Chinese soil classification system, the main soil types are alpine meadow soils (Chinese Soil Taxonomy Cooperative Research Group, Institute of Soil Science, Academic Sinica 1995), and in this region, these soils can freeze from August until April of the following year. The vegetation is a typical alpine meadow vegetation dominated by *Stipa purpura*, *Carex rigescens*, and *Kobresia humilis*. The length of the growing season is approximately 120–150 days, from April or May to September [41,42]. Yaks (*Bos grunniens*) are the predominant livestock and grazing pressure has increased over the past 50 years. During the growing season, the yaks are regularly grazed in fenced enclosures during the day, and are moved back to shelters were they spend the night, whereas during the non-growing season, yaks are often fed by herdsmen and remain within the shelters [5,42,43]. According to a local government report (http://www.zeku.gov.cn/contents/221/1136.html), 6187 km^2^ of grassland is available for grazing, which accounts for up to 92.49% of the entire area of Zeku County and has been fenced since the 1980s [44]. With the assistance of local administrators, herdsmen have lived in settlements in the area since 2000 [45].

### 2.2. Experimental Design and Sampling

We carried out the experiment at the end of August 2016, a period that coincided with the annual peak in biomass. Livestock density was calculated from the ratio of the number of livestock to the area of pasture. The experimental sites were grazed at three intensities, light (LG), moderate (MG), and heavy (HG), with respective stocking densities of 0.19, 0.53, and 1.42 yaks ha^−1^, the dry sheep equivalents of which are 0.87, 2.39, and 9.38 per hectare, respectively (Figure 1).

At each sampling site (10 m × 10 m), five plots (0.5 m × 0.5 m) were randomly selected for collecting above-ground biomass as well as dead leaves that were still attached to plants. An additional three plots (0.5 m × 0.5 m) were used for collecting below-ground biomass. The samples of plant material used for below-ground biomass analyses were initially rinsed in water to remove soil and debris. Both above- and below-ground biomasses were dried at 65 °C to constant weights, which were rounded to the nearest 0.1 g [46].

When collecting biomass at each sampling site, we also collected soil samples from three soil layers (0–10, 10–20, and 20–30 cm) using a corer (7.5 cm diameter). For each plot, five soil cores taken at the same depth were mixed together to provide a single composite soil sample. After visible roots and plant debris had been removed, the soil samples were air-dried at room temperature, and subsequently passed through a 2-mm sieve for soil organic carbon (SOC), total C (TC), total N (TN), and total P (TP) analyses. A soil profile (1 m in diameter and 1 m in depth) was excavated and soil samples from three soil layers (0–10, 10–20, and 20–30 cm) were collected using a cutting ring (100 cm^3^) for estimating bulk density (BD). BD was determined from the oven-dried soil mass [46] and soil water content (SWC) was determined gravimetrically at 105 °C for 24 h [47]. SOC content (SOCC) was determined using a volumetric K_2_Cr_2_O_7_ method [46]; TC was determined using the K_2_Cr_2_O_7_–H_2_SO_4_ method [48]; TN was determined using an automatic Kjeldahl analyzer (KDY-9830; Huawei Industrial Technology, Beijing, China) [49]; and TP was determined by digestion with H_2_SO_4_ and HClO_4_ [50].

### 2.3. Data Statistics and Analysis

Soil organic carbon stock (SOCS, t ha^−1^), soil total nitrogen stock (STNS, t ha^−1^), and soil total phosphorus stock (STPS, t ha^−1^) were calculated as follows [51]:

(1) $$Element_{stock}=element_{content}\times BD\times D$$

where element content is the soil organic carbon content (g kg^−1^), total nitrogen content (g kg^−1^), or total phosphorus content (g kg^−1^); *BD* is the soil bulk density (g cm^−3^); and *D* is the soil depth (m).

Both one- and two-way ANOVA analyses were conducted for the soil properties among the sampling sites with three grazing intensities and soil depths using IBM SPSS Statistics (ver. 18.0; IBM, New York, NY, USA) and figures presenting soil property data were generated using R (ver. 3.4.4; The R Foundation for Statistical Computing). Duncan’s multiple range test was conducted for post hoc comparisons. The effect of each variable was considered statistically significant at *p* < 0.05 (two-sided). Simple linear regression analyses were used to examine the relationships between the soil C, N, and P contents and ratios and grazing intensities and other environmental factors using R. SOCC values were used to calculate C:N and C:P ratios. The results are presented as the mean ± standard error (S.E.) of at least three replicates.

## 3. Results

### 3.1. Effect of Grazing on Biomass

Both above- and below-ground biomass decreased with an increase in grazing intensity among the sampling sites (Table 1). The lowest value of above-ground biomass (20.0 ± 1.6 g m^−2^) was recorded at the HG site, which was only 16.2% and 23.9% that of above-ground biomass at the LG and MG sites, respectively. Similarly, the lowest value of below-ground biomass recorded at the HG site (861.6 ± 116.5 g m^−2^), was approximately 70% of the values recorded at the LG and MG sites (Table 1).

### 3.2. Effect of Grazing on Soil Physical Properties

**Figure 2:**
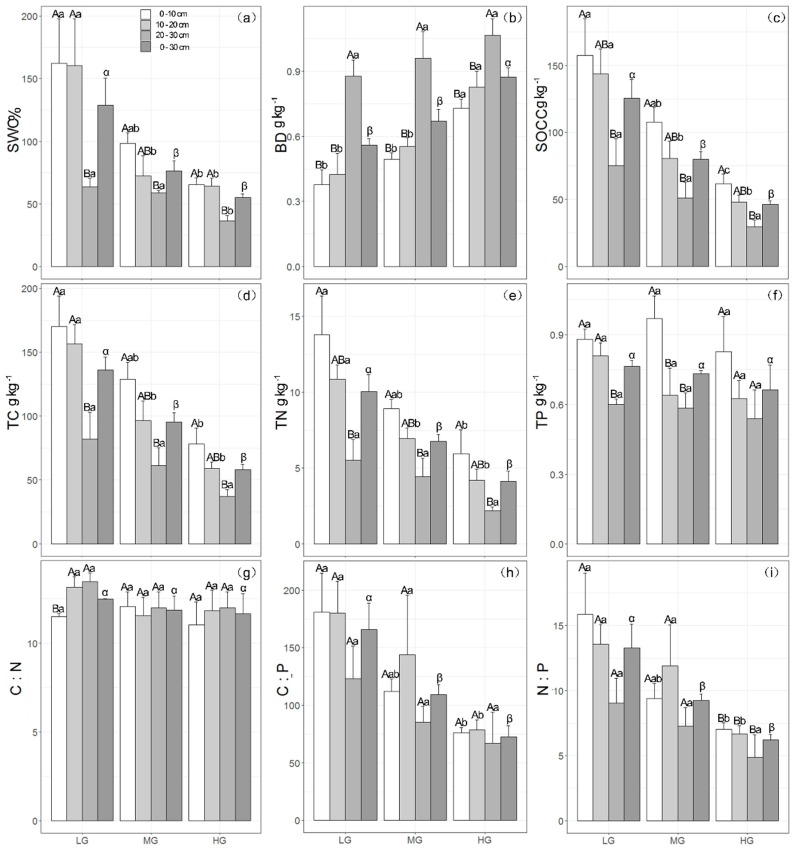
Distribution of soil properties at different grazing intensities and in different soil layers (one-way ANOVA analyses). (**a**) Soil water content (SWC); (**b**) bulk density (BD); (**c**) soil organic carbon content (SOCC); (**d**) total carbon (TC); (**e**) total nitrogen (TN); (**f**) total phosphorus (TP); (**g**) soil organic carbon content to total nitrogen (C:N) ratio; (**h**) soil organic carbon content to total phosphorus (C:P) ratio; and (**i**) total nitrogen to total phosphorus (N:P) ratio. Upper case letters indicate significant differences among three different soil layers (0–10, 10–20, 20–30 cm) at the same grazing intensity site; lower case letters indicate significant differences in the same soil layer (0–10, 10–20, 20–30 cm) among the three different grazing intensity sites [low (LG), moderate (MG), and heavy (HG)]; Greek letters indicate significant differences in the 0–30-cm soil layer among the three different grazing intensity sites; *n* = 27.

We observed decreases in soil water content (SWC) with an increase in grazing intensity and soil depth (Figure 2a). For example, the mean SWC values in the topmost two soil layers at the LG site were both more than twice those at the HG site (Figure 2a). For the topmost 30 cm of soil, SWC at the LG, MG, and HG sampling sites was 128.7 ± 21.9%, 76.6 ± 8.1%, and 55.3 ± 2.7%, respectively (Figure 2a). In contrast, bulk density (BD) increased with an increase in grazing intensity and soil depth, with the highest values being recorded at the HG site (Figure 2b), which were 0.7 ± 0.1, 0.8 ± 0.1, 1.1 ± 0.1, and 0.9 ± 0.1 g kg^−1^ in the 0–10, 10–20, 20–30, and 0–30 cm soil layers, respectively (Figure 2b).

### 3.3. Effect of Grazing on Soil Chemical Properties

Soil organic carbon content (SOCC), total carbon (TC), and total nitrogen (TN) decreased with an increase in grazing intensity and soil depth (Figure 2c–e). At the LG site, the values for SOCC in the topmost two soil layers (157.4 ± 27.5 g kg^−1^ and 143.8 ± 18.3 g kg^−1^ in the 0–10-cm and 10–20-cm layers, respectively) were significantly higher than that in the 20–30-cm soil layer (75.1 ± 20.2 g kg^−1^) (Figure 2c, *p* < 0.05). Moreover, the values of SOCC in the topmost two soil layers at the LG site were higher than those in the same layers at the HG site (Figure 2c), as were the values for TC and TN (Figure 2d,e). In addition, the maximum values of TC and TN in the 0–10-cm soil layer of the LG site were 170.1 ± 23.9 and 13.8 ± 2.6 g kg^−1^, respectively. For the topmost 30 cm of soil, the values for both TC and TN at the LG site were larger than those at the HG site with mean values of 115.7 ± 21.7 and 8.4 ± 1.8 g kg^−1^, respectively. However, there were no significant differences among total phosphorus (TP) values for the same soil layers at the three sampling sites (Figure 2f). The values of TP in the 0–30-cm soil layer at the LG, MG, and HG sites were 0.8 ± 0.0, 0.7 ± 0.0, and 0.7 ± 0.1 g kg^−1^, respectively (Figure 2f). Furthermore, we observed that TP decreased with soil depth at all sampling sites, with the lowest values of 0.6 ± 0.1 at LG, 0.6 ± 0.1 at MG, and 0.5 ± 0.1 kg^−1^ at HG site being recorded in the 20–30-cm soil layer (Figure 2f).

Although there were no significant differences in soil organic carbon stocks (SOCS) between the LG and MG sites in each of the 10-cm-interval soil layers (0–10, 10–20, and 20–30 cm), total SOCS in the topmost 30-cm of soil at the LG site was 31.7% higher than that at the MG site and also 72.6% higher than that at the HG site (Table 2). Similar to SOCS, the soil total nitrogen stock (STNS) of the 0–30-cm soil layer at the LG site (227.2 ± 22.2 t ha^−1^) was notably higher than that at the MG and HG sites (189.6 ± 10.0 and 151.4 ± 12.0 t ha^−1^, respectively) (Table 2). Although there were no significant differences among the 10-cm-interval soil layers (0–10, 10–20, and 20–30 cm) at the three sampling sites, the highest soil total phosphorus stock (STPS) in the topmost 30 cm of soil was found at the HG site, whereas the lowest was recorded at the LG site (Table 2).

### 3.4. Effect of Grazing on Stoichiometric Variation in Soil C, N, and P

There were no significant differences detected in the C:N ratio among the different grazing intensities and soil layers, with the mean values of 12.5 ± 0.0, 11.9 ± 0.8, and 11.6 ± 1.1 in the topmost 30 cm of soil at the LG, MG, and HG sampling sites, respectively (Figure 2g). However, both the C:P and N:P ratios decreased with an increase in grazing intensity (Figure 2h,i). The highest values for both C:P and N:P ratios (180.0 ± 33.9 and 15.9 ± 3.2, respectively) were recorded in the 0–10-cm soil layer at the LG site (Figure 2h,i), whereas the lowest values for both C:P and N:P ratios (67.1 ± 27.1 and 4.9 ± 1.7, respectively) were recorded in the 20–30-cm soil layer at the HG site (Figure 2h,i). C:P and N:P ratios at the LG site both decreased with an increase in soil depth. However, at the MG site, these ratios were highest in the 10–20-cm soil layer, although the differences were not statistically significant (Figure 2h,i). Although there were no obvious differences in C:P and N:P ratios among each of the three sampled soil layers at the LG and MG sites, values in the 0–30-cm soil layer at the LG site were considerably higher than those at the MG site (Figure 2h,i).

## 4. Discussion

The results obtained in this study revealed that a heavier grazing intensity decreases SOCC, TC, and TN (Figure 2c–e; *p* < 0.05), which is consistent with the findings of previous studies [9,52,53,54]. This indicates that heavier grazing intensities have the potential to decrease soil C and N contents, which could be attributable to the effects of livestock grazing and trampling. In this regard, previous studies have shown that grazing-induced declines in above-ground biomass can reduce vegetation coverage and increase soil water evaporation [15,55], soil erosion by wind [6], and decomposition of soil nutrients and litter [15]. These effects of grazing have been confirmed by previous studies demonstrating increases in soil C and N with an increase in above-ground biomass and ground cover following grazing exclusion [19,56]. Moreover, a grazing-induced decrease in below-ground biomass was shown to decrease C inputs from roots to soil [5]. Consequently, SOCC, TC, and TN are likely to decrease with an increase in grazing intensity. In other words, the higher the biomass production, the higher could be the soil SOCC and N content. This can probably be attributed to the fact that soil organic matter is the main source and pool of both soil C and N [57].

**Figure A1:**
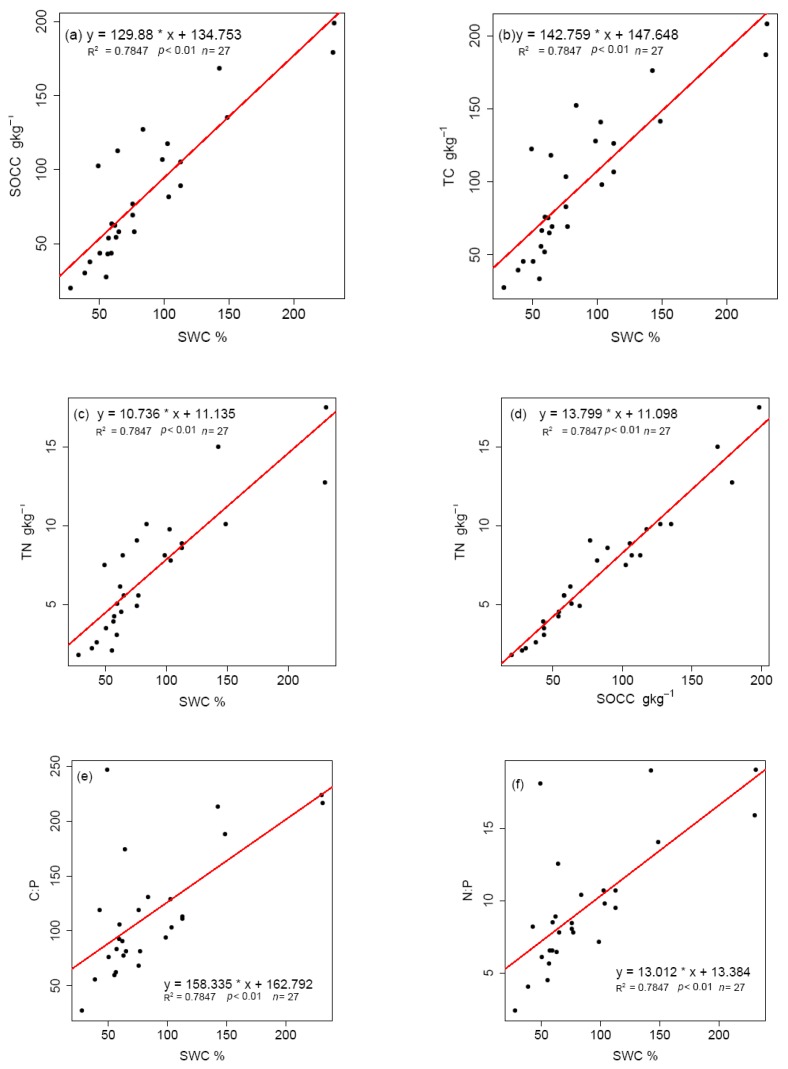
Regression between soil properties. (**a**) Soil organic carbon content (SOCC) and soil water content (SWC); (**b**) total carbon (TC) and SWC; (**c**) total nitrogen (TN) and SWC; (**d**) TN and SOCC; (**e**) SOCC to total phosphorus (C:P) ratio and SWC; and (**f**) TN to TP (N:P) ratio and SWC; *n* = 27.

Given that intensive grazing leads to lower biomass production, lower N and C contents would be expected. The observation that soil SOCC and N showed similar changes under the three different grazing intensities was confirmed by regression analysis indicating that soil C and N showed a significant positive relationship (Appendix A, Figure A1d; *p* < 0.01), whereas the C:N ratio remained stable (Figure 2g). This is consistent with the findings of a previous study in which C:N ratio values of 8.93 and 8.92 were recorded under light and heavy grazing, respectively [9].

However, the findings of some previous studies have indicated that heavy grazing can promote increases in soil C and N in the alpine meadow ecosystem (Table 1) [2,8]. This apparent paradox could be explained as follow. Firstly, Li et al. [8] reported that their HG site, which had the highest soil C and N levels, had a significantly higher soil water content (SWC), which is consistent with the findings of the present study indicating that there is a significant positive relationship between soil C and N and SWC (Appendix A, Figure A1a–c; *p* < 0.01). However, in our study, the highest SWC value was recorded at the LG site, which was subjected to the lowest grazing intensity. Although this contrasts with the observations reported by Li et al. [8], it is consistent with the findings of a study conducted by Zhang et al. [15]. Hence, grazing-induced effects on SWC could be one of the key factors influencing soil C and N accumulation. A higher SWC could be indicative of relatively low evaporation and higher soil tolerance to erosion, which tend to inhibit the decomposition of both soil C and N [5,15]. Accordingly, in the present study, we found that the soil contents of both C and N were higher under a lighter grazing intensity.

Secondly, both Gao et al. and Li et al. [2,8] have reported that a grazing-induced increase in root biomass could explain why heavier grazing intensity promotes higher soil C. Moreover, a meta-analysis conducted by McSherry and Richie indicated that an increase in the mass of fine and shallow roots in response to heavier grazing intensity could lead to an increase in SOCC [58]. However, our study showed that a heavy grazing intensity reduced below-ground root production (Table 1), which is consistent with the findings of previous studies by Zhang et al. [15] and Bai et al. [59]. Root biomass could thus be another key factor influencing soil C and N accumulation both in alpine meadow and semiarid grassland ecosystems [15,51,60]. This is in line with our observations in the present study, in which we demonstrate that SOCC, TC, and TN all increase with an increase in below-ground biomass from HG to LG (Figure 2c–e; Table 1). Further, grazing has been found to reduce the release of root exudates [61], which could result in a decrease in soil microbial C and N, and thereby have a potentially negative effect on soil C and N accumulation [62]. Consequently, the apparently paradoxical observation that heavy grazing can promote increases in soil C and N could be attributed to differences in SWC and root biomass in response to different grazing intensities. This in turn indicates the necessity of obtaining specific data (i.e., biomass, soil physical properties, and stocking densities) in order to compare the effects of grazing on soil C and N reported in the literature [63], and warrants further meta-analysis.

Given that the HG site was grazed by the highest density of livestock, this heavy grazing intensity could have altered the structure of the soil microbial community at this site; for example, by promoting lower fungal to bacterial ratios [15,64]. Such an alteration in the soil microbiota could increase soil C and N losses [6], consistent with the observations in the present study (Figure 2c–e). The high stock number would presumably have resulted in larger inputs of C, N, and P at the HG site via the deposition of livestock feces and urine, which would in turn promote increases in microbial biomass and activity, thereby enhancing native soil organic matter mineralization and leading to reductions in soil C, N, and P concentrations [5]. However, our observations tended to indicate that TP is insensitive to grazing intensity, as there were no pronounced differences in TP within comparable soil layers among the three sampling sites subjected to different grazing intensities (Figure 2f), which corresponds with the findings of Rui et al. [3]. This would imply that the input of P via livestock deposition does not increase soil TP content. Although TP decreased from the surface to deeper soil layers at each sampling site, the differences were not as pronounced as those for either TC or TN (Figure 2d–f), indicating that levels of TP are probably more stable under the present grazing management. Therefore, it is not surprising that both C:P and N:P ratios decreased with heavier grazing pressure, which is consistent with previous observations [1,59]. The higher C:P and N:P ratios recorded at the LG site compared with those at the HG site can possibly be attributed to the higher soil moisture at the former site, as indicated by the significant positive correlations between C:P and N:P ratios and SWC (Appendix A, Figure A1e,f, *p* < 0.01, respectively), which has also been reported at different altitudes in alpine ecosystems by Bing et al. [35]. Moreover, a lower SWC could enhance the decomposition of organic matter, which may have further led to a relatively higher TP and lower C:P and N:P ratios at the HG site. We also observed that at all the sampling sites, the soil C:P ratios were <200 (Figure 2h), indicating mineralization of soil P and that the cycling between inorganic and organic P was not stable [30], which is consistent with the pattern observed in intensively grazed systems in the UK [31].

The apparent stability of soil TP has similarly been observed in a study conducted on a meadow steppe, which showed no significant changes in TP between grazed and ungrazed sites [56]. The stability of soil P could be related to the fact that this P is derived mainly from the weathering of rocks rather than from organic matter decomposition, and that P has a low solubility in soil [65,66]. Consequently, the amount of soil organic matter derived from vegetation (i.e., litter, root exudates, and dead roots) appears to have less impact on soil P than on soil C and N. A further explanation for the apparent stability of P is that reduced fungal activity in response grazing limits P losses [15]. Given that mycorrhizal fungi are important participants in effective symbioses that enable vegetation to obtain soil P from otherwise unavailable forms [67], heavier grazing could inhibit the absorption of P by vegetation, thereby contributing to the maintenance of relatively stable soil P levels. Consequently, in the present study, soil TP showed no clear difference among the sites subjected to three different grazing intensities. This assumption is consistent with the findings of a previous study that indicated that soil P concentrations at two sites grazed under different stocking rates were both relatively stable over a 20-year period [68].

**Figure A2:**
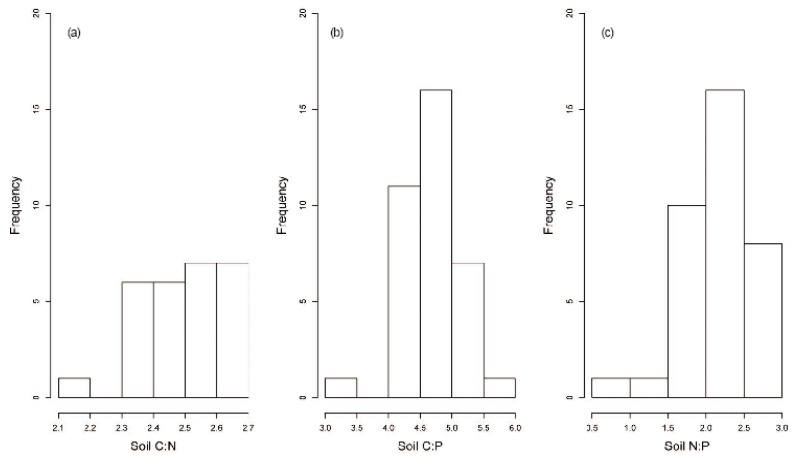
Frequency distribution of soil C, N, and P ratios. (**a**) Soil organic carbon content to total nitrogen (C:N) ratio; (**b**) soil organic carbon content to total phosphorus (C:P) ratio; and (**c**) total nitrogen to total phosphorus (N:P) ratio among sampling sites. The x-axis of the histogram is presented using a log(e) scale to highlight the lognormal distribution; *n* = 27.

In this study, we found that the soil C:N, C:P, and N:P ratios all followed a normal distribution, which is consistent with the pattern observed in previous studies [26,69]. Most of the C:N, C:P, and N:P ratios recorded were in the ranges 2.3–2.7, 4.0–5.5, and 1.5–3.0, respectively (Appendix A, Figure A2a–c). We also found that soil C:N:P ratios decreased with an increase in grazing intensity (Table 1). Previous studies have reported soil C:N:P ratios for terrestrial ecosystems at different scales [26,69]. When we compared these ratios with the ratios obtained in the present study, we found, for example, that the soil C:N:P ratio in surface soil layers at the LG site (175:15:1) is comparable with that of global soils (186:13:1) [26] and Chinese surface soils (134:9:1) [69], but higher than that recorded in other regions of the Qinghai-Tibetan Plateau (Table 1), which tend to be closer to the soil C:N:P ratios recorded at the HG site in the present study. These observations indicate that potential C storage is considerably larger at the LG site of our study region, compared with that reported in previous studies (Table 1) [1,2,8], and that heavy grazing leads to obvious losses in soil C and N in this region.

Similar to soil total nitrogen (STNS) and phosphorus (STPS) stocks, we detected no significant difference in soil organic carbon stocks (SOCS) in the 0–10- and 10–20-cm soil layers (Table 2), which is consistent with the findings of studies on other grazed grassland in semiarid steppe ecosystems [15]. One possible reason for this pattern could be that there is no significant difference in soil organic SOCC between the 0–10- and 10–20-cm soil layers at sites subjected to different grazing intensities (Figure 2c). Moreover, bulk density in the 10–20-cm soil layer was slightly higher than that in the 0–10-cm soil layer (Figure 2b). Therefore, there was little difference in the SOCS between the two topmost soil layers. However, for the topmost 30-cm of soil, both SOCS and STNS showed significant differences among the three assessed grazing intensities (Table 2, *p* < 0.05, respectively), which is a trend that has previously been observed at the eastern edge of the Qinghai–Tibetan Plateau [5]. This indicates that long-term grazing can decrease the amounts of C and N in the topmost 30-cm of soil. For STPS, the stocks in the topmost 30-cm of soil at the HG site were 36.5% higher than those at the LG site (Table 2), although we detected no significant differences among soil TP levels in each of the 10-cm-interval layers between the LG and MG sites (Figure 2f). These observations could be explained by the fact that soil bulk density at the HG site was considerably higher than that at the LG site (Figure 2b), which could lead to an overestimation of STPS at the HG site [70]. Furthermore, SOCS and STNS at the HG site might have been overestimated, although both SOCS and STNS at the HG site were still considerably lower than those at the LG site (Table 2). This does not necessarily indicate that heavy grazing pressure has contrasting effects on SOCS and STNS but might imply that the current heavy grazing management causes marked differences in the distribution of soil organic carbon and nitrogen in alpine meadows. These findings could help to raise awareness among local administrators as to the necessity to take appropriate action, such as a conversion from heavy and moderate grazing to light grazing under eco-compensation projects run by the government or non-governmental organizations, to ensure sustainable grassland management.

## 5. Conclusions

In this study, we limited our analysis of soil to within a depth range of 0 to 30 cm, which is assumed to be the range most influenced by both natural environmental factors and anthropogenic disturbance [71]. We found that soil properties, including stoichiometric variations in N and P, differed with respect to both grazing intensity and soil depth. In response to increases in grazing intensity and soil depth, SWC, SOCC, TC, and TN decreased, whereas BD increased. For each of the 10-cm intervals in the topmost 30 cm of soil, SOCS, STNS, and STPS showed no significant difference among sites subject to different grazing pressure. However, the stocks of C and N in the entire topmost 30 cm of soil decreased with an increase in grazing intensity. Although the soil C:N ratio was relatively stable at the sites subjected to different grazing intensities and showed no significant differences among the three sampled soil layers, heavy grazing intensity may have promoted a substantial degradation and loss of soil C. Furthermore, soil C:P and N:P ratios were clearly reduced in response to an increase in grazing intensity. Although soil TP decreased slightly with an increase in soil depth, there were no significant differences in TP in the same soil layer at the three sites with different grazing intensities, and we accordingly conclude that the current management of alpine meadows in the study area may have led to the mineralization of soil P. Although we should not ignore the fact that soil properties show inherent variability, there is no doubt that heavy grazing pressure has promoted the degeneration of alpine meadow in this study area. Accordingly, appropriate action, such as the initiation of eco-compensation projects, is deemed necessary to ensure sustainable grassland management. Furthermore, in order to enable a more thorough comparison with previous studies and provide directions for further studies on the effect of grazing on soil properties, it would be desirable to gain more information on in situ management in the study region (i.e., livestock density and history), as this would greatly enhance our understanding of ecosystem feedback to climate change and anthropogenic disturbance.

## Figures and Tables

**Table 1:** Vegetation biomass and soil properties of different surface soil layers under different grazing intensities on the Qinghai-Tibetan Plateau (mean ± S.E.), presenting a comparison of data obtained in the present study with data obtained previously in this region.

Study Sites	*n*	SL (cm)	GI (Yaks ha^−1^)	ABio (g m^−2^)	BBio (g m^−2^)	SOCC (g kg^−1^)	TN (g kg^−1^)	TP (g kg^−1^)	C:N:P	Ref.
Hongyuan, QTP	3	0–15	0.00			49.08	4.05	0.92	49:4:1	[1]
	3	0–15	0.71			67.85	5.93	1.06	68:6:1	
	3	0–15	1.20			59.03	5.03	0.98	59:5:1	
	3	0–15	1.58			54.89	4.72	1.02	55:5:1	
Hongyuan, QTP	5	0–30	1.20	521	1798	9795 *				[2]
	5	0–30	2.00	589	2482	10158 *				
	5	0–30	2.90	392	2923	11729 *				
Maqu, QTP	3	0–15	1.08	299.7	715.5	43.13	4.06	0.61	71:7:1	[8]
	3	0–15	1.36	231.5	1478.3	52.08	4.26	0.76	68:6:1	
	3	0–15	2.13	114.2	1899.4	60.83	5.70	0.80	76:7:1	
Zeku, QTP	3	0–10	0.19	123.3 ± 3.8	1219.3 ± 193.9	157.4 ± 27.5	13.8 ± 2.6	0.9 ± 0.0	175:15:1	This
	3	0–10	0.53	83.7 ± 9.9	1152.4 ± 203.4	107.7 ± 11.0	8.9 ± 0.6	1.0 ± 0.1	108:8:1	study
	3	0–10	1.42	20.0 ± 1.6	861.6 ± 116.5	61.6 ± 7.5	5.9 ± 1.6	0.8 ± 0.2	77:7:1	

Notes. S.E. indicates standard error; *n*, sample size of at least three replicates for ABio, SOCC, BBio, TN, and TP in each study. QTP, Qinghai-Tibetan Plateau; SL, soil layer; GI, grazing intensity; ABio, above-ground biomass; BBio, below-ground biomass; SOCC, soil organic carbon content; TN, soil total nitrogen; TP, soil total phosphorus; C:N:P, SOCC to TN to TP ratio. Grazing intensities were calculated from the ratio of the number of yaks to pastures area. If needed, the grazing intensities were normalized from Tibetan sheep to yaks (multiply by 0.2) among different studies, according to the Chinese standard (NY/T 635-2002) formulated the by Ministry of Agriculture of China (http://www.std.gov.cn/hb/search/stdHBDetailed?id=5DDA8BA2AC8218DEE05397BE0A0A95A7). * The unit of SOCC is g cm^−2^, according to Gao et al., 2007. The unit of SOCC was not converted from g cm^−2^ to g kg^−1^ because no soil bulk density data were presented by Gao et al., 2007.

**Table 2:** Soil characters of the topmost 30 cm of soil at sites with different grazing intensities (two-way ANOVA analyses). Values for soil organic carbon stocks (SOCS), soil total nitrogen stocks (STNS), and soil total phosphorus stocks (STPS) were obtained for the 0–10-cm, 10–20-cm, 20–30-cm, and 0–30-cm soil layers (SL) at lightly grazed (LG), moderately grazed (MG), and heavily grazed (HG) sites. Data represent the mean ± SE, *n* = 9.

SL (cm)	SOCS (t ha^−1^)			STNS (t ha^−1^)			STPS (t ha^−1^)		
	LG	MG	HG	LG	MG	HG	LG	MG	HG
0–10	56.4 ± 15.7de	53.3 ± 11.8de	44.3 ± 4.4de	61.4 ± 14.7DE	63.8 ± 14.1DE	55.9 ± 9.1DE	0.33 ± 0.10γ	0.47 ± 0.05γ	0.59 ± 0.12γ
10–20	57.7 ± 11.4de	43.6 ± 7.3de	39.0 ± 0.9de	63.9 ± 17.7DE	52.1 ± 8.9DE	47.9 ± 3.1DE	0.35 ± 0.17γ	0.36 ± 0.14γ	0.51 ± 0.12γ
20–30	64.6 ± 29.7d	46.3 ± 11.4de	30.8 ± 7.3e	70.2 ± 29.6D	55.4 ± 13.7DE	39.2 ± 8.1E	0.53 ± 0.08γ	0.55 ± 0.03γ	0.59 ± 0.27γ
0–30	208.7 ± 33.4a	158.5 ± 8.5b	120.9 ± 5.1c	227.2 ± 22.2A	189.6 ± 10.0B	151.4 ± 12.0C	1.26 ± 0.15β	1.47 ± 0.20αβ	1.72 ± 0.42α

Notes. Lower case letters, upper case letters, and Greek letters indicate significant differences in SOCS, STNS, and STPS in different soil layers among the three different grazing intensity sites, respectively.
